# Spatially Bandgap-Graded MoS_2(1−*x*)_Se_2*x*_ Homojunctions for Self-Powered Visible–Near-Infrared Phototransistors

**DOI:** 10.1007/s40820-019-0361-2

**Published:** 2020-01-18

**Authors:** Hao Xu, Juntong Zhu, Guifu Zou, Wei Liu, Xiao Li, Caihong Li, Gyeong Hee Ryu, Wenshuo Xu, Xiaoyu Han, Zhengxiao Guo, Jamie H. Warner, Jiang Wu, Huiyun Liu

**Affiliations:** 1 Department of Electronic and Electrical Engineering, University College London, Torrington Place, London, WC1E 7JE UK; 2 School of Energy, Soochow Institute for Energy and Materials Innovations, and Key Laboratory of Advanced Carbon Materials and Wearable Energy Technologies of Jiangsu Province, Soochow University, Suzhou, 215006 People’s Republic of China; 3 London Centre for Nanotechnology, University College London, London, WC1H 0AH UK; 4 Institute of Fundamental and Frontier Sciences, University of Electronic Science and Technology of China, Chengdu, 610054 People’s Republic of China; 5 Department of Materials, University of Oxford, Parks Road, Oxford, OX1 3PH UK; 6 Department of Chemistry, University College London, 20 Gordon St, Bloomsbury, London, WC1H 0AJ UK; 7 Department of Chemistry, The University of Hong Kong, Pokfulam Road, Hong Kong, People’s Republic of China; 8 Zhejiang Institute of Research and Innovation, The University of Hong Kong, Qingshan Lake SciTech City, Hangzhou, People’s Republic of China

**Keywords:** Transition metal dichalcogenides, Graded bandgaps, Homojunctions, Phototransistors, Self-powered

## Abstract

Ternary transition metal dichalcogenide alloys with spatially graded bandgaps are an emerging class of two-dimensional materials with unique features, which opens up new potential for device applications. Here, visible–near-infrared and self-powered phototransistors based on spatially bandgap-graded MoS_2(1−*x*)_Se_2*x*_ alloys, synthesized by a simple and controllable chemical solution deposition method, are reported. The graded bandgaps, arising from the spatial grading of Se composition and thickness within a single domain, are tuned from 1.83 to 1.73 eV, leading to the formation of a homojunction with a built-in electric field. Consequently, a strong and sensitive gate-modulated photovoltaic effect is demonstrated, enabling the homojunction phototransistors at zero bias to deliver a photoresponsivity of 311 mA W^−1^, a specific detectivity up to ~ 10^11^ Jones, and an on/off ratio up to ~ 10^4^. Remarkably, when illuminated by the lights ranging from 405 to 808 nm, the biased devices yield a champion photoresponsivity of 191.5 A W^−1^, a specific detectivity up to ~ 10^12^ Jones, a photoconductive gain of 10^6^–10^7^, and a photoresponsive time in the order of ~ 50 ms. These results provide a simple and competitive solution to the bandgap engineering of two-dimensional materials for device applications without the need for p–n junctions.

**Electronic supplementary material:**

The online version of this article (10.1007/s40820-019-0361-2) contains supplementary material, which is available to authorized users.

## Highlights

- Due to the Se composition and thickness gradient within single MoS_2(1−*x*)_Se_2*x*_ domains, the bandgap of MoS_2(1−*x*)_Se_2*x*_ is gradually tuned from 1.83 to 1.73 eV.
- The homojunction phototransistors at zero bias deliver a photoresponsivity of 311 mA W^−1^, a specific detectivity up to ~ 10^11^ Jones, and an on/off ratio up to ~ 10^4^.
- The biased devices yield a champion photoresponsivity of 191.5 A W^−1^, a specific detectivity up to ~ 10^12^ Jones, and a photoconductive gain of 10^6^–10^7^.

**Electronic supplementary material:**

The online version of this article (10.1007/s40820-019-0361-2) contains supplementary material, which is available to authorized users.

## Graphical abstract

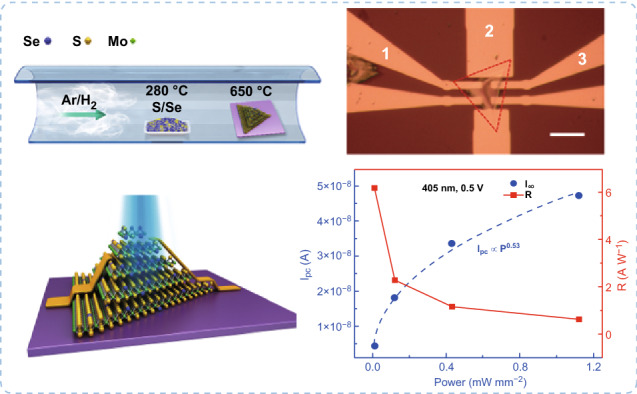

## Introduction

Two-dimensional (2D) materials exhibit new properties due to the unique atomic thin-layered structure. The rich variety of 2D materials provides many different electrical, optical, and chemical properties. Heterostructures with “on-demand” properties can be prepared by stacking different 2D sheets via the van der Waals (dW) force, and the strict requirement in lattice match facing conventional thin films can be relaxed. Additionally, compared with traditional materials used for photodetection (e.g., MCT and III–V compound semiconductors), these new materials have the advantages of high absorption coefficient, mechanical robustness, and easy synthesis, which promise low-cost, lightweight, and high-performance detectors.

2D layered transition metal dichalcogenides (TMDs), MX_2_ (M = Mo, W; X = S, Se, Te), have attracted substantial attention for prospective applications, including field-effect transistors, photodetectors, lasers, memories, etc., due to the tunable bandgap not only from indirect to direct but also across the range from visible to near-infrared (NIR) spectral region [1–11]. To achieve high-performance optoelectronic and photonic devices and extend the already fascinating properties of the constituents, further bandgap engineering has played a crucial role. This has been mostly realized by creating vertically stacked or in-plane heterostructures, such as exfoliated-restacked MoTe_2_/MoS_2_ and epitaxially grown WS_2_-MoSe_2_ [12–16]. Recently, in situ controlling the alloy composition within a single domain of ternary TMDs, such as Mo_1−*x*_W_*x*_S_2_, WS_2*x*_Se_2(1−*x*)_, and MoS_2(1−*x*)_Se_2*x*_, has been introduced to tune the bandgaps, which demonstrated an alternative to modulate bandgaps and provided more design flexibility [17–20]. However, most of the as-grown ternary alloys show a fixed elemental composition, which requires further processing (e.g., wet or dry transferring) to prepare hetero-/homojunctions for devices. The spatially bandgap-graded alloys avoid the drawback by ideally forming a homojunction that possesses continuously tuned band structures, without abruption as in the case of heterojunctions, and results in a lower adjacent barrier height and more energy-friendly [21–24]. Additionally, the in-plane homojunction can deliver a strong built-in electric field with optimum spatial bandgap grading.

Due to the spatially graded bandgap within a single-alloy domain, such homojunctions provide a novel platform for versatile optoelectronic devices. Grading of stoichiometry induced graded bandgap has been introduced to zero-dimensional (0D) material based solar cells for energy harvesting [25, 26]. Bandgap-graded one-dimensional (1D) alloys enabled by composition gradient have also been systemically investigated and showed competitive properties to conventional 1D materials [27–29]. It is still limited to study the spatially bandgap-graded 2D TMDs within a single domain [30, 31]. Although the fabrication of lateral composition-graded 2D materials without thickness variation is rather rewarding, it is a challenge to form desired heterostructures (e.g., p–n junction) for electronics and optoelectronics.

Here, we demonstrated spatially bandgap-graded 2D homojunction devices with p–n diode-like current rectification and photovoltaic response. The homojunctions, stemming from graded composition and thickness together, showed gate-tuned electrical and/or optical properties and enabled visible–NIR and self-powered phototransistors. Gradually selenized MoS_2(1−*x*)_Se_2*x*_ alloys with thickness gradient were synthesized on a single substrate using a one-step and controllable chemical solution deposition (CSD) method. The structural asymmetry of the graded MoS_2(1−*x*)_Se_2*x*_ homojunction produced an intrinsic build-in potential, and hence diode-like characteristics. Such graded homojunctions may relax the need for p–n junctions and serve as alternative building blocks for advanced electronics and optoelectronics.

## Experimental Section

### Material Synthesis

Ammonium molybdate, sulfur, and selenium powders were used as Mo, S, and Se sources, respectively. TheSiO_2_/Si substrates (300 nm) were pretreated by piranha solution for good hydrophilicity. For smooth few-layer alloy growth, 0.12 g KOH and 0.05 g ammonium molybdate were dissolved into the 5 mL deionized water to obtain the hydroxide-assisted aqueous solution for smooth MoS_2(1−*x*)_Se_2*x*_ nanosheets with uniform thickness, while we used 0.06 g KOH instead for N/P alloy growth. Then the precursors were spin-coated onto the cleaned SiO_2_/Si substrates. Subsequently, an alumina boat loaded with several as-processed SiO_2_/Si substrates was placed at the heating center of the tube furnace, and another boat loaded with 20 mg sulfur and 20 mg selenium powders was placed at the upstream site. In an Ar/H_2_ (5% H_2_) atmosphere, the two zones were independently heated up to 650 and 280 °C, respectively. After keeping at 650 °C for 30 min, the furnace was then naturally cooled down to room temperature.

### Material Characterizations

Room-temperature ADF-STEM imaging was performed using a JEOL ARM200F at 200 kV located at the David Cockayne Center for Electron Microscopy (DCCEM) within the Department of Materials at the University of Oxford. Imaging conditions used a 30-µm CL aperture with a convergence semi-angle of 22.5 mrad and a beam current of 35 pA. The acquisition angles for these images were 72.8–271 mrad. Dwell time per pixels was typically 32 µs.

The morphology of the MoS_2(1−*x*_)Se_2*x*_ domains was characterized using scanning electron microscopy (SEM, FEI Scios, 15 kV), and the thickness was measured by AFM (Bruker Dimension Icon, tapping mode). X-ray photoelectron spectroscopy (XPS, Thermo Fisher ESCALAB 250Xi) was used to characterize the chemical composition of alloys. High-resolution transmission electron microscopy (HRTEM) imaging was performed on a field emission TEM (FEI Tecnai F20, 200 kV); selected-area electron diffraction (SAED) measurements were performed on a TEM operating at 120 kV (FEI Tecnai T12). Raman and micro-PL spectra/mapping were collected with a confocal Raman spectrometer (Horiba Jobin–Yvon HR Evolution) using a 532 nm laser as the excitation source. The laser spot size was 1 mm, and the laser power on the sample surface was kept below 60 mW.

### Device Characterizations

The electrical and photoresponsive measurement was conducted at the ambient condition, using a Keithley 4200 semiconductor parameter analyzer equipped with a white light and laser sources. The lasers are unfocused with a spot size of ~ 1.5 mm in diameter. The power density for the 405, 650, and 808 nm lasers was 120, 15.8, and 110 µW mm^−2^, respectively.

The photoresponsivity was calculated based on Eq. 1:

1 $$ R = \frac{{I_{\text{pc}} }}{PS} $$

here *R*, *I*_pc_, *P*, and *S* are defined as photoresponsivity, net photocurrent (*I*_pc_ = *I*_laser_ − *I*_dark_), laser power density, and active area of a photodetector, respectively.

Assuming that shot noise from the dark current is the major contributor to the total noise, the specific detectivity (D^*^) of photodetectors can be estimated from Eq. 2:

2 $$ D^{*} = R\sqrt {\frac{S}{{2qI_{\text{dark}} }}} $$

where *q* is the elementary charge.

### Theoretical Details

All the calculations were carried out based on density functional theory (DFT), implanted in Vienna ab initio Package (VASP) [32] The projector-augmented wave method was employed to describe the core region with a cutoff of 500 eV [33]. The PBE functional was employed for describing the electronic exchange–correlation [34]. Spin-orbital coupling and dipole correction along the out-of-plane direction were considered [35]. Gamma-centered 25 × 25 × 1 k-points were used for sampling monolayer of MoS_2_ and MoSe_2_.

## Results and Discussion

**Fig. 1:**
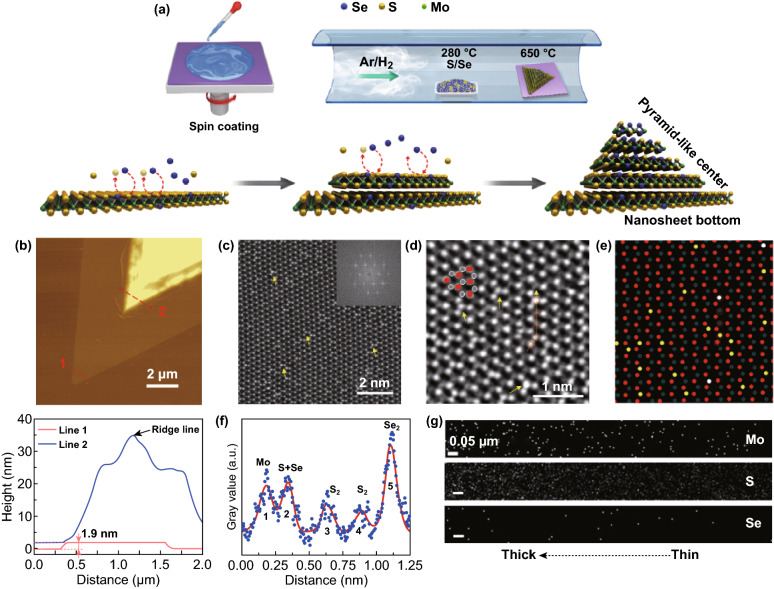
Synthesis and characterization of MoS_2(1−*x*_)Se_2*x*_ alloys. **a** Synthesis schematics of the spatially bandgap-graded MoS_2(1−*x*_)Se_2*x*_ alloys with a N/P structure, illustrating S atoms were partially substituted by Se atoms during the growth. The green, yellow, and blue balls denote Mo, S, and Se atoms. **b** AFM image of the N/P homojunction domain and AFM height profiles along lines 1 and 2. **c** ADF-STEM image and digital diffractogram (the inset) of the edge of the MoS_2(1−*x*)_Se_2*x*_ N/P domain. **d** Further magnified ADF-STEM image, showing different contrast levels and Se substitution. The red and gray balls represent Mo and S atoms, respectively, and the typical hexagonal rings are illustrated by them. The five atoms along the yellow arrow are red circled. **e** Schematic map of different atom distribution, illustrating the sites where the single and double S were substituted by Se. The red, dark gray, yellow, and white dots represent Mo atoms, S_2_ units, S + Se units, and Se_2_ units, respectively. The five atoms along the arrow in **d** are also circled. **f** Intensity profile of the five atoms along the yellow arrow labeled in **d**. **g** EDS maps from STEM of Mo, S, and Se atoms about the red box in Fig. S2d. The thickness increases from the right to the left side and the scale bar is 50 nm. (Color figure online)

MoS_2(1−*x*)_Se_2*x*_ alloys were grown using a controllable CSD method on SiO_2_/Si substrates. As depicted in Fig. 1a (the upper), cleaned SiO_2_/Si substrates were spin-coated with a precursor solution dissolved with KOH and ammonium molybdate and then loaded into a home-built tube furnace. In the Ar/H_2_ (5% H_2_) atmosphere, the mixed S/Se powders (1:2 wt%) at the upstream gas site and the as-processed substrates at the downstream site were independently heated up to 280 and 650 °C within 60 min, respectively. Noteworthy, sulfurization tends to take place at 650 °C or even lower, while at least 750 °C for full selenization [36–38]. As a result, after keeping the substrates at 650 °C for 30 min then followed by natural cooling down to the room temperature, MoS_2(1−*x*)_Se_2*x*_ alloys with partial S atoms replaced by Se atoms (the lower in Fig. 1a) were crystallized on SiO_2_/Si substrates. Through changing the concentration of KOH, we can selectively obtain either few-layer MoS_2(1−*x*)_Se_2*x*_ nanosheets with uniform thickness or MoS_2(1−*x*)_Se_2*x*_ homojunction domains with varied thickness. Figure S1a displays an SEM image of few-layer MoS_2(1−*x*)_Se_2*x*_ nanosheets with identical color contrast, suggesting the uniform thickness. In Fig. S1b, the high-resolution TEM and selected-area electron diffraction (SAED) images demonstrated the high crystallinity and hexagonal symmetry, respectively. In addition, both the Raman (Fig. S1c) and X-ray photoelectron spectroscopy (XPS) spectra (Fig. S1d, e) validate the successful incorporation of Se atoms into MoS_2_. As a result, the atomic percentage of Se was estimated to be ~ 10%, indicating few-layer MoS_2(1−*x*)_Se_2*x*_ nanosheets possessed fixed elemental composition when thickness unchanged.

In contrast, the SEM image of MoS_2(1−*x*)_Se_2*x*_ homojunction domains in Fig. S1f identifies the surface ridge lines marked by the red arrows, implying a pyramid-like configuration with varied thickness. An atomic force microscopy (AFM) image in Fig. 1b (upper) depicts the surface topography of a homojunction domain. The different color contrast associated with varied thickness and clear grain boundaries were observed, suggesting a stacked configuration composed of a thin edge and a thick center (brighter color). Figure 1b (lower) illustrates the height profiles along the line 1 and line 2 marked in the AFM image. It determines the uniform thickness of 1.9 nm corresponding to the three-layer (3L) nature for the edge while gradually thickened and pyramid-like configuration for the multilayer center. Evidently, the highest point in the blue height profile originates from the ridge lines observed in Fig. S1f. Namely, the homojunction domain can be viewed as a similar nanosheet/pyramid (N/P) structure with thickness grading, whose schematic diagram is shown in the bottom-right corner in Fig. 1a [7]. Such N/P structure was further validated by the homojunction domains selected for device fabrication in Fig. 3d. To determine the specific atomic distribution in homojunction domains, annular dark-field scanning transmission electron microscopy (ADF-STEM) characterization was performed. The ADF-STEM (Fig. 1c) and the inset of digital diffractogram images, obtained from the edge with uniform thickness of the N/P domain, show the well-aligned crystal lattice and hexagonal symmetry, respectively, suggesting the high crystallinity of the N/P structure. It was reported that Se atoms exhibited higher intensity than S atoms during the ADF-STEM imaging [20], so it can be inferred that the locations with different contrast (e.g., marked by the yellow arrows) in Fig. 1c were the sites where Se atom incorporation took place in MoS_2_. The further magnified ADF-STEM image (Fig. 1d) clearly identifies the atomic hexagonal rings of MoS_2_ and the atoms with different contrast at the sites originally corresponding to S atoms, such as the yellow arrow pointed ones, indicating the S atoms were substituted by Se atoms. To examine the characteristics of atomic substitution quantitatively, the gray values of the five atoms (red circled) along the yellow path labeled in Fig. 1d were extracted and plotted in Fig. 1f, in which the changed intensity stems from the different atomic kinds and amount. The five peaks are obtained successively as the yellow arrow passes by the five circled atoms. Thus, the peaks corresponding to Mo and S atoms were resolved according to the atomic arrangement of MoS_2_. Compared to the referenced Mo atoms (peak 1), S_2_ units (peak 3 and 4) with no Se replacement display lower gray intensity. Peak 2 with similar intensity to Mo atoms was assigned to the units of S + Se with the top S replaced by Se, while peak 5 exhibiting higher intensity than Mo atoms was attributed to the Se_2_ units with double S replaced by Se, consistent with the previous work [20]. On the basis of particular intensity, the schematic diagram (Fig. 1e) extracted from Fig. 1d, straightforwardly maps the corresponding distribution of the single and double Se substitution sites, presented with the yellow and white dots, respectively. These atomic incorporation characteristics were further verified by another N/P domain, as shown in Fig. S2a–c. Moreover, EDS was employed to study the spatial distribution of Mo, S, and Se atoms from the atomically thin edge to the thick center in the N/P homojunction alloys. An area with thickness grading was selected. Figure 1g, obtained from the selected area framed in Fig. S2d, illustrated more Mo, S, and Se signal was detected (white dots) when getting closer from the thin edge to the thick center (from the right to the left). This was resulted from the layer-by-layer accumulated elemental signal as thickness continuously increased, confirming the thickness grading.

Afterward, XPS was employed to determine the respective composition changing trend of Mo, S, and Se based on the dominant binding energy peaks as thickness changed. In Fig. S2e, dot 1–4 with gradually increased thickness from the edge to the center, judged by the optical contrast, were selected for elemental content analysis within a single N/P domain. As illustrated and estimated in Fig. S3, from the thin site (dot 1) to the thick site (dot 4), Se-3d% raised from ~ 18.47 to ~ 21.72% gradually, while Mo-3d% kept around ~ 33.5% and S-2p% accordingly dropped from ~ 47.97 to ~ 44.50% progressively. Evidently, XPS results revealed the elemental content changing trend complied with the stoichiometry shown in MoS_2(1−*x*)_Se_2*x*_. They also demonstrated the formation of a spatial Se composition gradient and resulted in S composition gradient when thickness gradually varied.

**Fig. 2:**
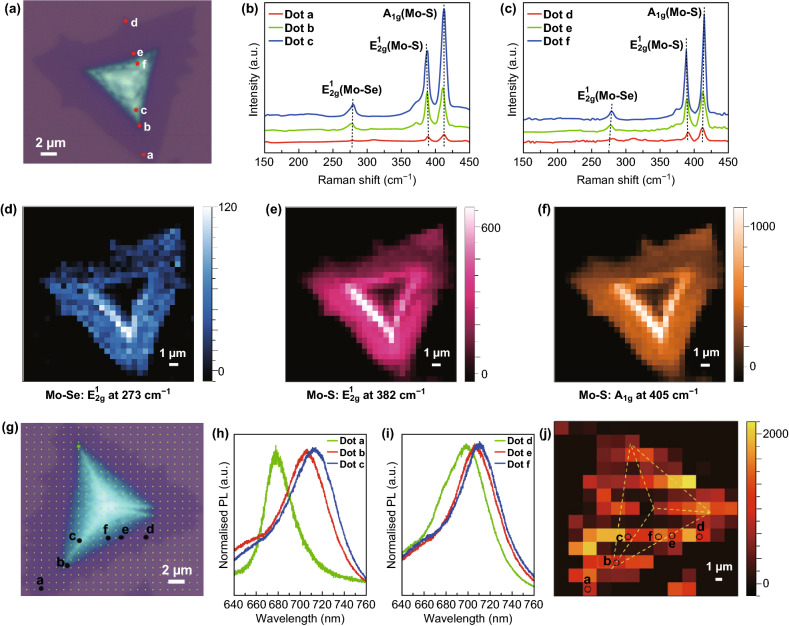
Optical properties of the spatially bandgap-graded MoS_2(1−*x*_)Se_2*x*_ alloys. **a** Optical microscopy image of a N/P MoS_2(1−*x*)_Se_2*x*_ nanoflake for Raman spectroscopy study. **b**, **c** Position-dependent Raman spectra measured from dot *a* to dot *c* and from dot *d* to dot *f*. **d**–**f** Raman intensity maps of the domain in **a** for the *E*_2*g*_^1^ (Mo–Se), *E*_2*g*_^1^ (Mo–S), and *A*_1*g*_ (Mo–S) modes. **g** Optical microscopy image of a N/P MoS_2(1−*x*)_Se_2*x*_ nanoflake for micro-PL measurements. **h**, **i** Position-dependent micro-PL spectra obtained from dot *a* to dot *c* and from dot *d* to dot *f*. **j** Micro-PL intensity map for the N/P domain shown in **g**. The black circled pixels correspond to the black dots marked in **g**. The area defined by the yellow dashed lines represents the central pyramid domain. (Color figure online)

To probe the spatial composition and thickness gradient induced impacts on optical properties of the alloy homojunctions, we conducted Raman and micro-PL studies under 532 nm laser excitation, using a confocal Raman spectrometer. Figure 2b shows the position-dependent Raman spectra of the N/P domain displayed in Fig. 2a. In the Raman spectrum attained from dot ***a***, the wavenumber difference between the two dominant eigen peaks from MoS_2_ (in-plane *E*_2*g*_^1^ mode and out-of-plane *A*_1*g*_ mode) is 23 cm^−1^, indicating the 3L configuration of the edge area (consistent with the AFM height analysis shown in Fig. 1b) [39, 40]. From dot ***a*** (at the thin edge) to dot ***c*** (at the thick center), this value is continuously increased to 26 cm^−1^ while *E*_2*g*_^1^ mode redshifts and *A*_1*g*_ mode blueshifts. Upon the reported studies, the redshift of the *E*_2*g*_^1^ mode should be mainly originated from the varied S% and increased number of layers [39–41]. For the *A*_1*g*_ mode, the blueshift is attributed to the stronger electron coupling with increased electron concentration according to the previous study, which could be induced by the changed concentration of S and Se in the thick center of the N/P [42]. It is worth noting that for the pristine binary MoSe_2_, the in-plane *E*_2*g*_^1^ mode is redshifted and its intensity decreases as the number of layers increases [43]. In contrast, from dot ***a*** to dot ***c***, the MoSe_2_-like *E*_2*g*_^1^ mode (at ~ 273 cm^−1^) observed in our ternary N/P domain shows a slight blueshift (Fig. 2b) or unnoticeable shift (Fig. S4b) but obvious rising intensity with increased thickness, which can be thereby caused by the Se% variation. Additionally, such phenomena of Raman peak shift and enlarged intensity from dot ***a*** to dot ***c*** were also observed in the single crystalline ternary MoS_2(1−*x*_)Se_2*x*_ alloy when the Se content was continuously increased [17, 19, 44]. Thus, it was reasonable to deduce that the features of position-dependent Raman spectra were presumably resulted from the Se composition gradient from dot ***a*** to dot ***c***, which was further confirmed by the measurement from dot ***d*** to dot ***f*** (Fig. 2c) and observed in an entirely different 2D flake (Fig. S4a–c).

Figure 2d–f shows the corresponding Raman intensity maps of the *E*_2*g*_^1^ (Mo–Se), *E*_2*g*_^1^ (Mo–S), and *A*_1*g*_ (Mo–S) modes, respectively. Obviously, the Raman peak intensity of the three Raman-active modes increases as the layer number increases from the edge to the center, which can be attributed to the Se/S composition and thickness gradient. With continuous increase in the thickness, the optical interference is involved with the coupling of the excitation laser and the emitted Raman scattering near the center [40, 45]. As a result, the Raman peak intensity gradually reduces when moving close to the center for all the three phonon modes. Therefore, the Raman intensity variation features of the three Raman-active modes confirm the N/P configuration with graded thickness. Additionally, Fig. 2h–j shows the position-dependent micro-PL spectra and intensity maps of the N/P domain displayed in Fig. 2g. The thickness of the single crystalline binary MoS_2_ has an obvious impact on its emission wavelength. Once the thickness increased, the micro-PL spectra slightly redshifted with a small degree < 20 meV but drastically dropped intensity [10, 11]. In contrast, as shown in Fig. 2h, i, from dot ***a*** (at the thin edge) to dot ***c*** (at the thick center) (from dot ***d*** to dot ***f***), the peak redshifted obviously from 677 (695) to 715 nm (713 nm) (corresponding to 100 and 45 meV energy variation, respectively). This peak shift should be attributed to more introduction of Se from the edge to the center [17, 19, 30, 31]. Moreover, as shown in Fig. 2j, the peak intensity underwent unapparent change (e.g., from dot ***a*** to dot ***c***) or even strengthened (e.g., from dot ***d*** to dot ***f***) as thickness increased. Such irregular intensity variation of exciton peaks can stem from spatial strain inhomogeneity, affecting exciton states and populations and thus PL intensity [46]. The strain inhomogeneity was likely induced by the spatial grading of Se composition and thickness in our cases. In addition, almost no PL phenomenon was observed for the very central (thick) areas, owing to the local field effect [11]. Therefore, the features of PL peak shift and intensity changing can arise from the spatial composition together with thickness gradient. The micro-PL results were further identified in a different N/P 2D flake (Fig. S5). According to the XPS, Raman, and PL results, it was logical to assume that the thin edges of N/P domains can be more MoS_2_-like, while the thick centers tended to be more MoSe_2_-like. This suggested that the corresponding Fermi levels can be gradually different from the edges to the centers, which was further determined by the theoretical calculation below.

**Fig. 3:**
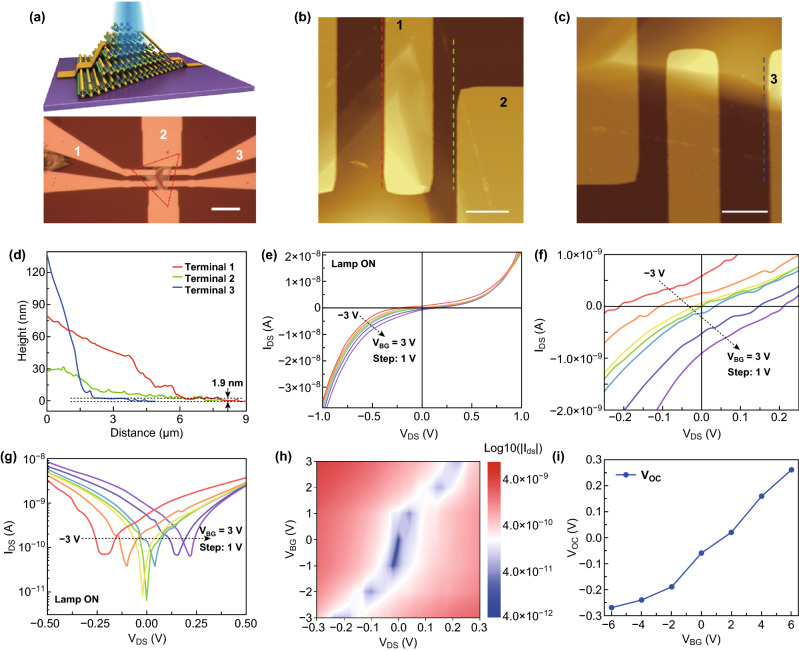
Characteristics of the alloy homojunction phototransistors. **a** A 3D schematic and an optical image of the phototransistor after EBL patterning and metallization. The scale bar is 20 μm. The triangular area defined by the red dashed lines represents the base of the flake which is a 3L MoS_2(1−*x*)_Se_2*x*_ nanosheet. **b**, **c** AFM images showing the three metal contacts of the phototransistor deposited at different positions at the N/P domain. The scale bar is 2 μm. **d** Height profiles along the three dashed lines marked in **b** and **c** for the three metal contacts. **e** Drain–source current versus drain–source voltage (*I*_DS_ − *V*_DS_) curves of the phototransistor measured between contact 1 (drain) and 3 (source) under lamp illumination. **f**, **g** Enlarged view of (**e**) nearby the origin point in linear and logarithmic scale, respectively. **h** The minimum *I*_DS_ map as a function of *V*_DS_ and *V*_BG_. The current is set in absolute value. **i** The dependence of *V*_OC_ on *V*_BG_, extracted from Fig. S7. (Color figure online)

The presence of spatial Se composition and thickness gradient is able to lead to the formation of a bandgap-graded homojunction and thus a built-in electric field. This is desirable for 2D devices as accurate spatial doping cannot be easily obtained in 2D nanosheets. Figure 3a shows the 3D view of a phototransistor based on the bandgap-graded 2D homojunction. The source and drain electrodes were fabricated by e-beam lithography (EBL) and then e-beam evaporation of Ti/Au (10 nm/50 nm). The optical microscopy image of the selected N/P homojunctions after metallization is also shown in Fig. 3a. Figure 3d identifies the height profiles of the three metal contacts along the dashed lines marked in the AFM images of Fig. 3b, c, which reveal the different height profiles. Although the thickness of each contact increased to different heights, they all started from the bottom 1.9 nm nanosheet and illustrated the thickness grading, which was accompanied with Se composition gradient according to the above material characterization analysis.

Figure 3e shows the output characteristics of the device measured between contacts 1 and 3 (drain and source, respectively, marked in Fig. 3a, distance > 3 µm) under illumination. The back gate voltage was varied from − 3 to 3 V. A rectifying behavior modulated by gate voltage was illustrated, of which the irregular dependence on gate voltage has also been reported in different systems. It can be attributed to the built-in electric field [47, 48]. The zoomed-in linear and logarithmic plots of output curves nearby the origin point are plotted in Fig. 3f, g, respectively, clearly showing a gate-tunable photovoltaic effect. With the gate voltage changing from the negative to the positive value, a consistently shifted *V*_OC_ from − 0.22 to 0.22 V was obtained. Figure 3h, presenting the map of the drain-source current (the smallest current corresponds to the *V*_OC_) in absolute value versus drain–source bias and back gate voltage, further highlights the dependence of *V*_OC_ on the back gate voltage. This behavior was confirmed on the same device by measuring between contacts 2 and 3 (drain and source distance < 1 µm). Similarly, as displayed in Fig. S6, the output curves obtained under illumination delivered similar reverse current level but larger rectification ratio of forward to reverse current, which should be owing to the lower homojunction barriers when illuminated. Notably, the *V*_OC_ can be continuously tuned from − 0.27 to 0.26 V as the back gate voltage varied from − 6 to 6 V (Figs. 3i and S7). When the back gate voltage is zero, *V*_OC_ of − 60 mV is obtained. In comparison, a device based on a non-graded 2D with fixed elemental composition and uniform thickness shows neither rectifying feature nor photovoltaic effect under illumination (Fig. S8a–c). Accordingly, this gate-modulated photovoltaic effect is reasonably attributed to the built-in electric field in the spatially bandgap-graded homojunctions, and the homojunctions were induced by spatial Se composition and thickness gradient in the N/P structure. The photovoltaic effect observed here is stronger and more sensitive (larger *V*_OC_ and modulated by smaller back gate voltage) than the previously reported ones, which were based on perovskites/2D WSe_2_ heterojunctions and MoSe_2_ homojunctions enabled by varied thickness [48, 49]. It is further verified by the self-powered photoresponse properties below.

**Fig. 4:**
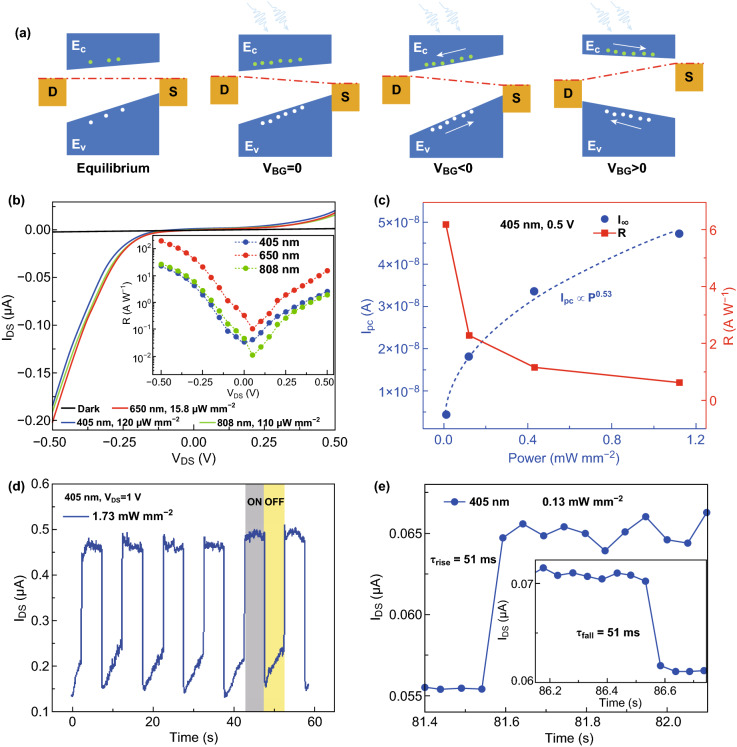
Mechanism and photoresponsive performance of the self-powered phototransistors. **a** Schematic band diagrams of the homojunctions at different states, respectively. The red dashed line represents the Fermi level. The green and white dots denote electrons and holes, respectively. For each band diagram, the Se composition increases from the left side to the right side. **b** Photocurrent curves measured under 405, 650, and 808 nm laser illumination, compared with the dark current curve. The bias-dependent photoresponsivity for the three lights is displayed in the inset. **c** Power density-dependent photocurrent and photoresponsivity for the device when illuminated by 405 nm light at V_DS_ = 0.5 V. **d** Dynamic on/off photoresponse of the phototransistors excited by 405 nm light. The biased voltage is 1 V. **e** Zoom-in views of the time-resolved 405 nm photoresponse at *V*_DS_ = 1 V, identifying the typical rise and fall time. (Color figure online)

Based on the calculated band structures of pristine MoS_2_ and MoSe_2_ (Fig. S9a, b), the bandgap-graded alloy possesses a type II-like development band alignment (Fig. S9c) along with the doping ratio [21, 22]. Considering the potential offset caused by the asymmetry of the N/P configuration, equivalent band diagrams are schematically shown in Fig. 4a. When illumination is present under zero gate voltage, the photogenerated carriers were separated and collected under the built-in electric field, resulting in modulated Fermi level and a nonzero potential. When applied a negative gate voltage, the built-in electric field is amplified, leading to larger *V*_OC_. Reversibly, by applying a positive gate voltage, the built-in electric field is compensated until being changed to a reversed direction, leading to the reversed sign of *V*_OC_.

Subsequently, photoresponse characteristics of the phototransistor (measured between contact 1 (drain) and 3 (source)) were studied when illuminated by visible–NIR lights at ambient conditions. As depicted in Fig. 4b, the dark current shows almost linear and symmetric curve passing through the origin point, while the photocurrent exhibits evident rectifying behavior, delivering an on/off ratio of ~ 10^2^ at *V*_DS_ = − 0.5 V. The bias-dependent photoresponsivity (*R*) was plotted in the inset. The phototransistor delivered the different *R* of 23.2, 191.5, and 26.2 A W^−1^ when excited by 405, 650, and 808 nm lasers at − 0.5 V bias, respectively. Remarkably, high detectivity (D^*^) up to ~ 10^12^ Jones was achieved for 650 and 808 nm lights. Figure 4c displays the dependence of photocurrent (*I*_pc_) and *R* on the incident power density (*P*) of the 405 nm laser. The scattered dots of *I*_pc_ were fitted using the power law, showing a nonlinear trend. This non-unity exponent indicates the presence of carrier traps and their involvement in electron–hole generation and recombination processes in homojunctions. Opposite to the changing trend of *I*_pc_, *R* decreases with power density of the incident laser increasing. The time-resolved photoswitching characteristics of the device illuminated by the 405 nm laser at *V*_DS_ = 1 V are displayed in Fig. 4d, illustrating dynamic stability and reproducibility. Figure 4e shows the typical rise time of 51 ms and fall time of 51 ms. In addition, the carrier lifetime $\tau_{\text{L}}$ on the order of 10^−1^–10^0^ s level can be extracted by fitting Eq. 3 (Fig. S10) [50]:

3 $$ I\left( t \right) = \alpha {\text{e}}^{{ - t/\tau_{\text{L}} }} $$

here *α* is a fitting parameter. This long carrier lifetime suggested that electrons can recirculate in the homojunction channels for multiple times following each photoexcited electron–hole generation. As a result, a very high photoconductive gain (*G*) was estimated to be on the order of 10^6^ ~ 10^7^ from Eq. 4:

4 $$ G = \frac{{\tau_{\text{L}} }}{{\tau_{\text{transit}} }} $$

here $\tau_{\text{transit}}$ denotes carrier transit time that was deduced in supporting information (Fig. S8d and Eq. S2).

**Fig. 5:**
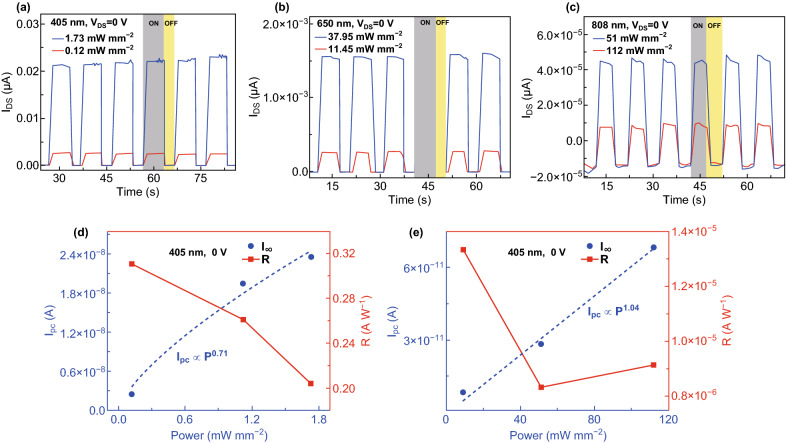
Self-powered photoswitching characteristics of the devices and power density-dependent photocurrent and photoresponsivity. **a-c** Self-powered on/off photoresponse characteristics of the device illuminated by 405, 650, and 808 nm lights. **d**, **e** Dependence of photocurrent and photoresponsivity on the incident power density of 405 and 808 nm lights, respectively. The external bias is zero

Given the strong built-in electric field aforementioned, the phototransistors are expected to work without external bias. Figure 5a–c shows the self-powered photoresponse characteristics of the device illuminated by 405, 650, and 808 nm lasers, respectively. Notably, an on/off ratio up to ~ 10^4^ and D^*^ up to ~ 0^11^ Jones were obtained for the 405 nm laser (1.73 mW mm^−2^). When the device was self-powered, *I*_pc_ and *R* as functions of *P* of the 405 and 808 nm lasers are displayed in Fig. 5d, e, respectively. Even at zero bias, the device still yielded *R* of 311 mA W^−1^ for the 405 nm wavelength. *I*_pc_ shows linear dependence on *P*, indicating that the total number of photogenerated carriers increased proportionally with the absorbed photon number, particularly under 808 nm illumination. Overall, the figures of merit of our phototransistors were competitive or even superior, particularly possessing the self-powered working mode, to those of mostly reported photodetectors based on 2D or multilayer TMDs, 2D layered semiconductors, and artificial heterojunctions under similar conditions [51]. Their performance comparison is shown in Table 1.

**Table 1:** Performance parameters of selected photodetectors based on 2D materials

Description	Responsivity (A W^−1^)	Specific detectivity (Jones)	On/off ratio	Response time (ms)	Spectral range	References
Monolayer MoS_2_ phototransistors	7.5 × 10^−3^	–	10^3^	50	Visible	[52]
Multilayer MoS_2_ phototransistors	0.11	10^11^	10^6^	~ 1000	Visible–near-infrared	[53]
Few-layer MoS_2_ phototransistors	6.3 × 10^−5^	4.2 × 10^8^	10^3^	20	UV–visible	[54]
MoSe_2_ photodetectors	93.7	–	~ 10	400	Visible	[55]
Monolayer MoSe_2_ phototransistors	–	–	10^3^	< 25	Visible	[56]
Graphene–MoS_2_ hybrid phototransistors	10	–	< 1.2	280	Visible	[57]
Few-layer MoS_2_-PbSe QDs photodetectors	1.9 × 10^−6^	–	–	250	Near-infrared	[58]
MoS_2_/black phosphorus photodetectors	22.3	3.1 × 10^11^	10^4^	0.015	Visible–near-infrared	[59]
Few-layer MoS_2_/WS_2_ photodetectors	2.3	–	~ 4	> 1000	Visible	[60]
MoS_2(1−*x*)_Se_2*x*_ phototransistors	191.5	~ 10^12^	10^4^	~ 50	Visible–near-infrared	This work

## Conclusions

In summary, we have fabricated visible–NIR and self-powered phototransistors based on N/P homojunctions (spatially composition and thickness graded MoS_2(1−*x*)_Se_2*x*_) using a one-step and cost-effective CSD approach. ADF-STEM was used to map the distribution of Se dopants and resolve the single and double Se substitution sites. Systematic material characterizations were performed to confirm the gradient of Se composition and thickness within the individual flakes. According to the shifted peaks in micro-PL spectra, the bandgaps of the alloys were continuously tuned from 1.83 to 1.73 eV across the homojunctions, leading to the formation of an internal built-in electric field. Consequently, the strong and sensitive gate-tunable photovoltaic effect was measured, enabling the photodetectors to work without external bias. The highest photoresponsivity of 311 mA W^−1^, D^*^ of ~ 10^11^ Jones and on/off ratio up to ~ 10^4^ (illuminated by 405 nm laser) were attained when self-powered operating. The biased devices delivered the champion performance when excited by the lights ranging from 405 to 808 nm: a photoresponsivity of 191.5 A W^−1^, a specific detectivity up to ~ 10^12^ Jones, a very high photoconductive gain of 10^6^–10^7^, and a fast photoresponse time in the order of ~ 50 ms. Overall, the realization of the high-performance visible–NIR phototransistors based on such spatially bandgap-graded TMD homojunctions opens up new scope of bandgap engineering and device design.

## Electronic supplementary material

Below is the link to the electronic supplementary material.

Supplementary material 1 (PDF 914 kb)
