# Supplementary material for: Spatially Bandgap-Graded MoS2(1−x)Se2x Homojunctions for Self-Powered Visible–Near-Infrared Phototransistors
Source: Nanomicro Lett. 2020 Jan 18;12:26. doi: 10.1007/s40820-019-0361-2 (PMC7770748; doi:10.1007/s40820-019-0361-2)
Supplement: Supplementary file 1 — Supplementary material 1 (PDF 914 kb) [file 40820_2019_361_MOESM1_ESM.pdf]

Nano-Micro Letters

Supporting Information for

Spatially Bandgap-Graded $MoS_{2(1-x)}Se_{2x}$ Homojunctions for Self-Powered Visible-Near-Infrared Phototransistors

Hao Xu[1, *, #], Juntong Zhu[2, #], Guifu Zou[2, *], Wei Liu[1, 3], Xiao Li[1], Caihong Li[4], Gyeong Hee Ryu[5], Wenshuo Xu[5], Xiaoyu Han[6], Zhengxiao Guo[6, 7, 8], Jamie H. Warner[5], Jiang Wu[1, 4, *], Huiyun Liu[1]

[1]Department of Electronic and Electrical Engineering, University College London, Torrington Place, London WC1E 7JE, UK

[2]School of Energy, Soochow Institute for Energy and Materials Innovations, and Key Laboratory of Advanced Carbon Materials and Wearable Energy Technologies of Jiangsu Province Soochow University, Suzhou 215006, People's Republic of China

[3]London Centre for Nanotechnology, University College London, London WC1H 0AH, UK

[4]Institute of Fundamental and Frontier Sciences, University of Electronic Science and Technology of China, Chengdu 610054, People's Republic of China

[5]Department of Materials, University of Oxford, Parks Road, Oxford OX1 3PH, UK

[6]Department of Chemistry, University College London, 20 Gordon St, Bloomsbury, London WC1H 0AJ, UK

[7]Department of Chemistry, The University of Hong Kong, Pokfulam Road, Hong Kong, People's Republic of China

[8]Zhejiang Institute of Research and Innovation, The University of Hong Kong, Qingshan Lake SciTech City, Hangzhou, People's Republic of China

[#]Hao Xu and Juntong Zhu contributed equally to this work

*Corresponding authors. E-mail: xuhaosilvia@gmail.com (Hao Xu); zouguifu@suda.edu.cn (Guifu Zou); jiangwu@uestc.edu.cn (Jiang Wu)

Supplementary Figures

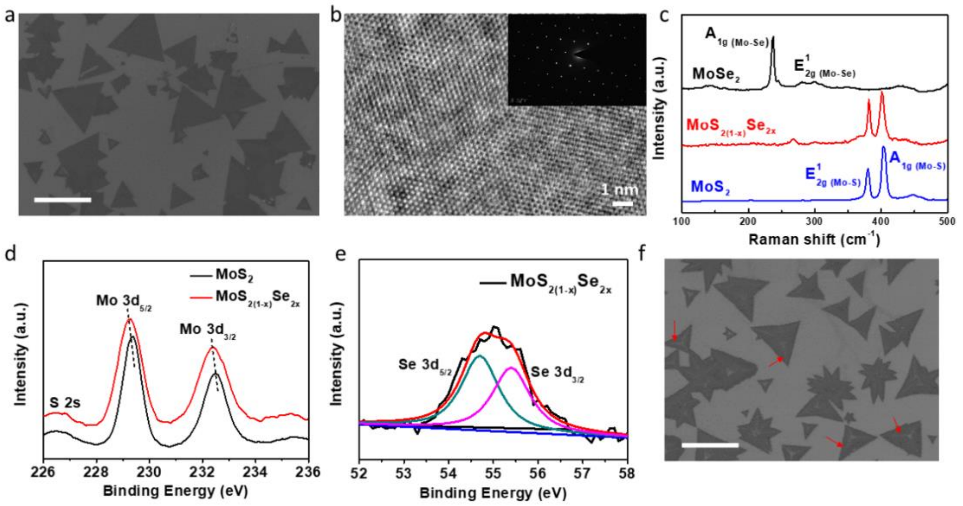

Fig. S1 Material characterization results of the as-grown $MoS_{2(1-x)}Se_{2x}$ alloys on SiO_2/Si substrates. (**a**) SEM image shows the smooth few-layer $MoS_{2(1-x)}Se_{2x}$ nanosheets with uniform

thickness, synthesized by using the aqueous solution dissolved with 0.12 g KOH and 0.05 g ammonium molybdate. The scale bar is 30 μm. (**b**) Both the selected-area electron diffraction (SAED) and high-resolution transmission electron microscopy (HR-TEM) results indicate that the as-grown smooth few-layer $MoS_{2(1-x)}Se_{2x}$ nanosheets are highly crystalline. (**c**) Comparison of the Raman spectra among the pure $MoSe_2$, pure MoS_2 and smooth few-layer $MoS_{2(1-x)}Se_{2x}$ alloy nanosheets, confirming the successful incorporation of Se. (**d, e**) XPS scans show the Mo, S and Se binding energies of the selected smooth few-layer $MoS_{2(1-x)}Se_{2x}$ nanosheets, and the x value was estimated to be ~0.1.[1] (**f**) An SEM image shows the nanosheet/pyramid (N/P) alloys with varied thickness in dominant, grown by using the aqueous solution dissolved with 0.06 g KOH and 0.05 g ammonium molybdate. The ridge lines are indicated by the red arrows. The scale bar 30 μm

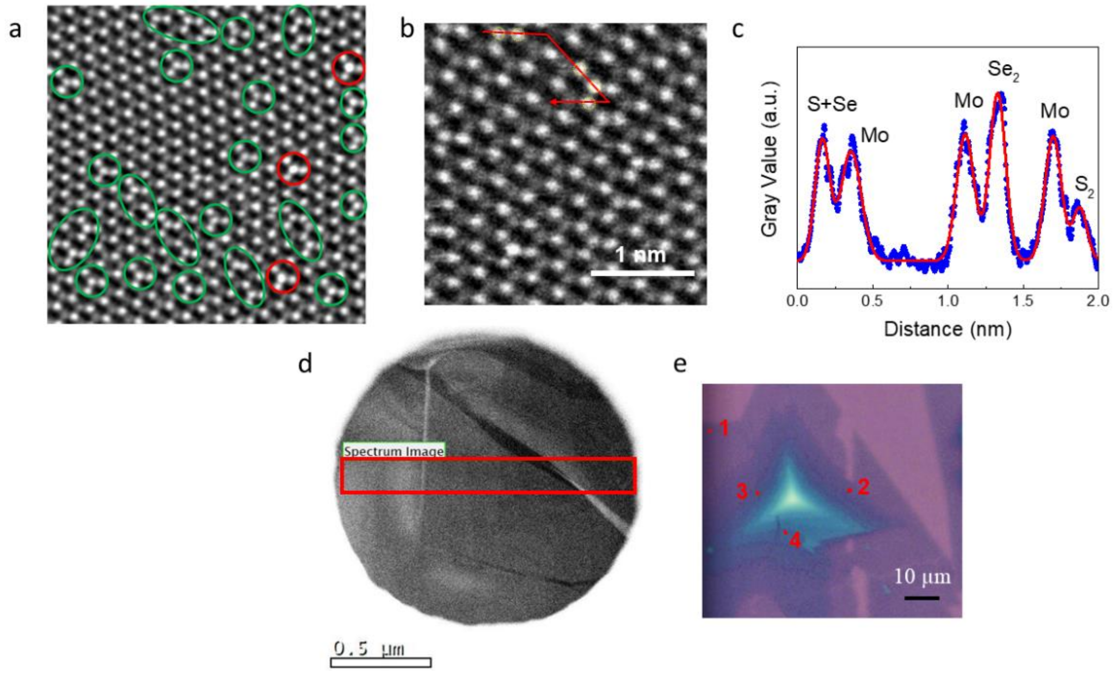

Fig. S2 (**a**) The annular dark field scanning transmission electron microscopy (ADF-STEM) image of a different $MoS_{2(1-x)}Se_{2x}$ alloy. The green and red circled sites correspond to single and double Se substitution, respectively. (**b**) The further magnified ADF-STEM image. The five atoms along the red arrow are yellow circled. (**c**) Intensity profile of the five atoms along the red arrow labelled in (**b**). Similar to **Fig.** 1f, the S_2 units show the lowest intensity while the S + Se units have the similar intensity to Mo atoms. The Se_2 units, caused by the double S replaced, have the largest intensity. (**d**) The spectrum imaging (SI) survey image. The framed zone was the selected area for energy dispersive spectroscopy (EDS) measurement. The thickness increases from the right to the left area. (**e**) Optical microscopy image of the N/P homojunction domain for XPS study

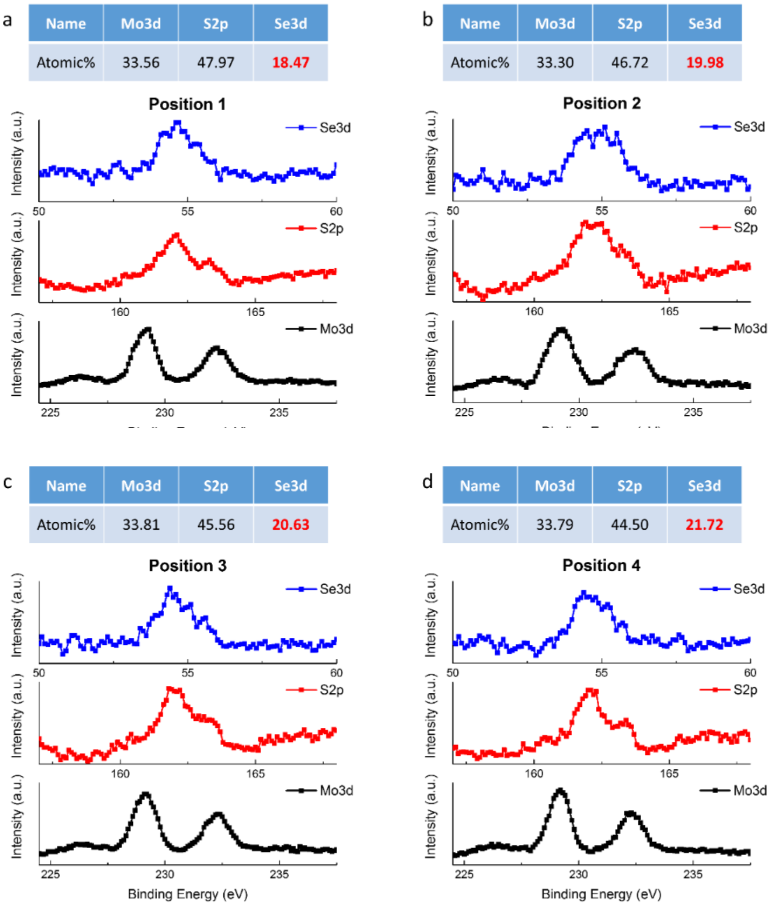

Fig. S3 XPS spectra and the dominant elemental bond proportion, obtained from the four positions marked in **Fig.** S2e

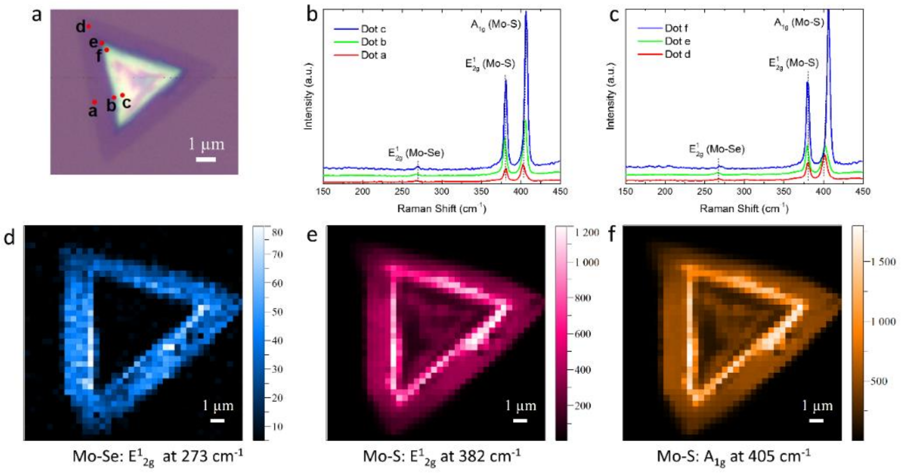

Fig. S4 Position-dependent Raman spectroscopy and intensity maps of the N/P bandgap-graded alloy domain. (**a**) Optical microscopy image of the N/P homojunction domain. (**b, c**) Position-dependent Raman spectra obtained from dot *a* to *c* and from dot *d* to *f*, respectively, in which the $MoSe_2$-like mode (at ~ 273 cm^{-1}) shows unobvious peak shift. The frequency difference between the two dominant eigen-peaks from MoS_2 (in-plane E^1_{2g} mode and out-of-

plane A_{1g} mode) increases from 23 to 26 cm^{-1} from the edge to the center, with E^1_{2g} mode red-shifted and A_{1g} mode blue-shifted. (d – f) Raman intensity maps of the domain shown in (a) for the E^1_{2g} (Mo-Se), E^1_{2g} (Mo-S) and A_{1g} (Mo-S) modes, respectively. The peak intensity of the three phonon modes continuously strengthens as the layer number increases from the edge to the center. The intensity in the thick center is decreased due to the optical interference as explained in the main text.

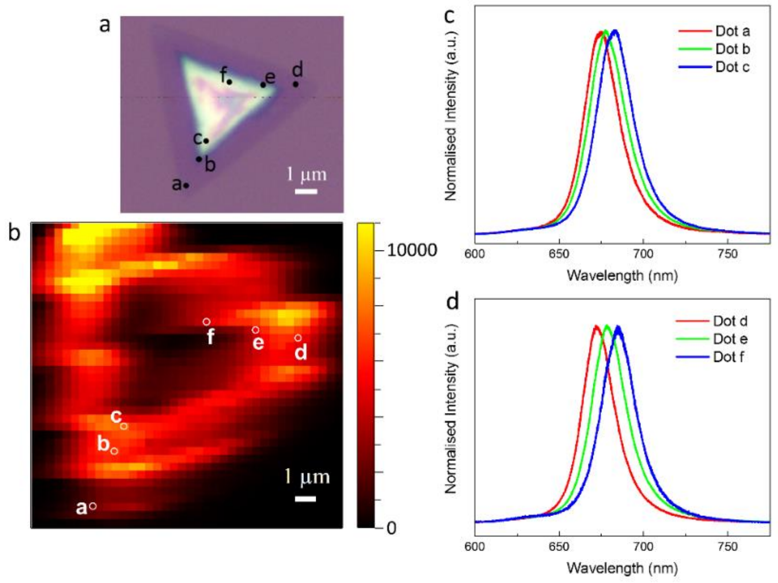

Fig. S5 Position-dependent micro-PL spectroscopy and intensity map of the same domain as the one shown **Fig.** S4a. (**a**) Optical microscopy image of the N/P domain. (**b**) Micro-PL intensity mapping. (**c, d**) Position-dependent micro-PL spectra obtained from dot *a* to *c* and from dot *d* to *f*, respectively, showing obvious red-shifts. From dot *a* to *c* (from dot *d* to *f*), the peak red-shifts obviously from 675 (673) to 686 nm (687 nm).

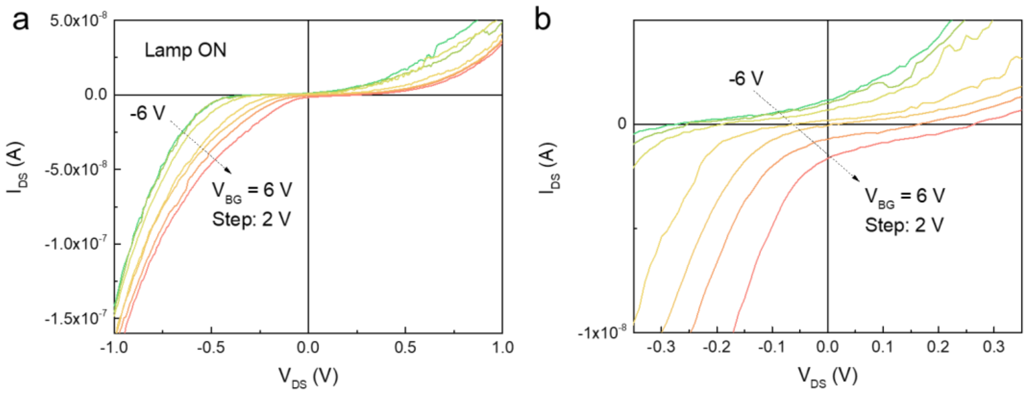

Fig. S6 Drain-source current-voltage (J-V) characteristics of the alloy homojunction phototransistor. (**a**) Drain-source J-V output curves of the phototransistor between contacts 2 (drain) and 3 (source) under illumination. (**b**) Zoom-in J-V output curves in linear scale. The back gate voltage V_{BG} changed from -6 to 6 V

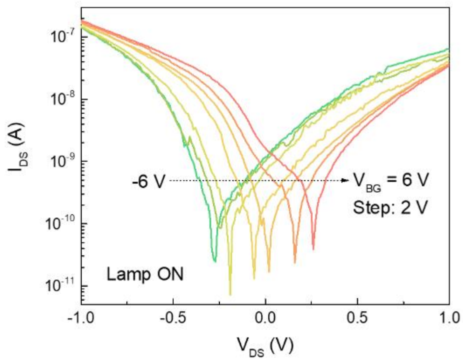

Fig. S7 Drain-source J-V output curves of the phototransistor between contacts 2 (drain) and 3 (source) under illumination in log scale. The back gate voltage V_{BG} changed from -6 to 6 V.

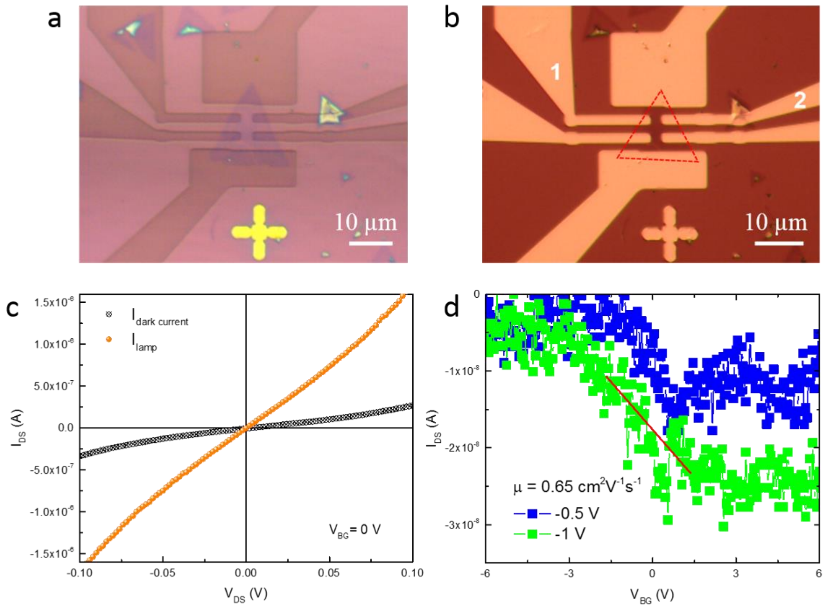

Fig. S8 Optical microscopy image of a phototransistor based on a non-graded 2D flake (**a**) before and (**b**) after metallization. (**c**) Drain-source J-V characteristics without any gate voltage with and without illumination. (d) Drain-source current vs back gate voltage characteristics. The electron mobility μ of 0.65 cm^{2}V^{-1}s^{-1} was deduced by Eq. S1 [S2] :

$$\mu = \frac{dI_{DS}}{dV_{BG}} \frac{L}{WC_i V_{DS}} \qquad \text{(S1)}$$

Here, L (3.4 μm), W (2 μm) and C_i (1.15 × 10^{-4} F m^{-2}) represent the channel length, the channel width and the capacitance between the channel and the back gate, respectively. $t_{transit}$ is defined as Eq. S2 [S3]:

$$\tau_{transit} = \frac{L^2}{\mu V_{DS}} \qquad \text{(S2)}$$

Here, μ and L denote the electron mobility and the channel length, respectively. Thus, the transient time was calculated to be ~ 10^{-7} s.

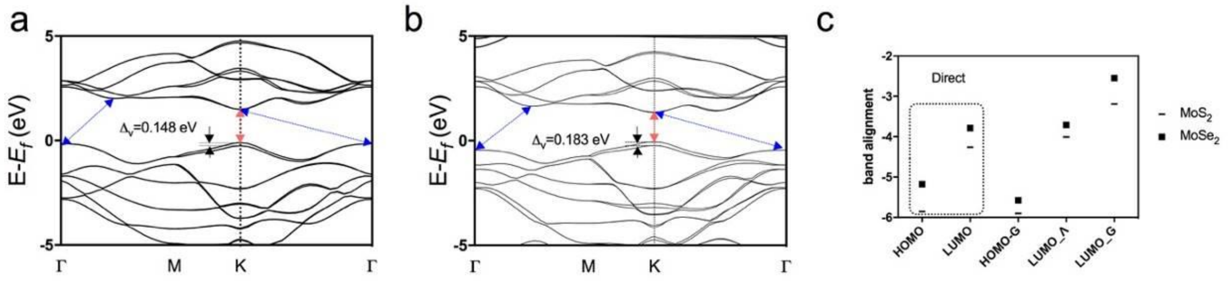

Fig. S9 (**a, b**) Band structures of MoS_2 and $MoSe_2$, respectively. Both transition metal dichalcogenides (TMDs) exhibit significant spin-orbital coupling. The arrows represent the possible exciton transfer ways. The Fermi level was set to zero. (**c**) The band alignment of MoS_2 and $MoSe_2$. The vacuum level set to zero

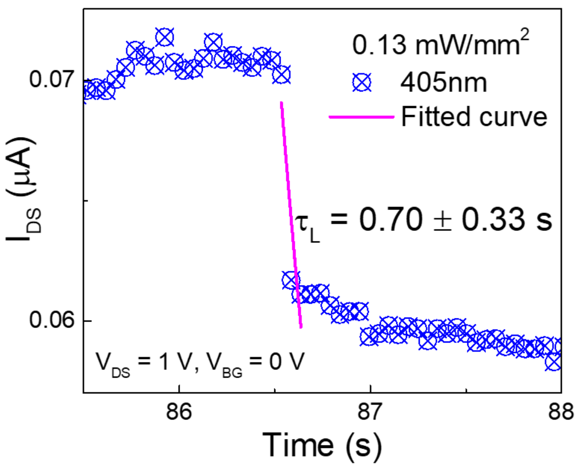

Fig. S10 The lifetime of the carriers photogenerated by 405 nm light, extracted from the fitted curve

Supplementary References

[S1]Yang, L.; Fu, Q.; Wang, W.; Huang, J.; Huang et al., Large-area synthesis of monolayered $MoS_{2(1-x)}Se_{2x}$ with a tunable band gap and its enhanced electrochemical catalytic activity. Nanoscale **7**, 10490–10497 (2015). https://doi.org/10.1039/C5NR02652K

[S2]Radisavljevic, B.; Radenovic, A.; Brivio, J.; Giacometti, V.; Kis, A. Single-layer MoS_2 transistors. Nat. Nanotechnol. 6, 147–150 (2011). https://doi.org/10.1038/nnano.2010.279

[S3]Huo, N.; Konstantatos, G. Ultrasensitive All-2D MoS_2 phototransistors enabled by an out-of-plane MoS_2 pn homojunction. Nat. Commun. **8**, 1–6 (**2017**). https://doi.org/10.1038/s41467-017-00722-1
